# The Endocrine Glands in Relation to Dental Nutrition and Caries*Read before the Philadelphia Dental Society, March 27, 1923.

**Published:** 1923-07

**Authors:** Charles E. deM. Sajous

**Affiliations:** Philadelphia, Pa., Professor of Applied Endocrinology, University of Pennsylvania Graduate Medical School


### THE ENDOCRINE GLANDS IN RELATION TO DENTAL NUTRITION AND CARIES*

BY CHARLES E. deM. SAJOUS, M. D., LL. D., Sc. D.,

PHILADELPHIA, PA.

Professor of Applied Endocrinology, University of Pennsylvania Graduate Medical School

The treasures revealed recently in a Pharaoh's tomb recalled in a measure what I experienced over two decades ago, after collecting and analyzing the data gathered for my treatise on the internal secretions—the first book published on the subject. The treasures beheld, however, did not all, as you might surmise, belong to the endocrinological group, but mainly to general medical knowledge. Briefly, the work had enabled me to appreciate our wealth in actual assets in all branches of medicine. Much of it lay hidden, however, in the dust of oblivion and all of it in confusion. Co-ordination, analysis and synthesis, by filling, if possible, the many obvious gaps, might, it was thought, render service. It was in the latter capacity that endocrinology seemed to afford promise. It appeared capable of playing the part of a great white light comparable to the electric light cast into the many compartments in which the Egyptian treasures lay hidden. While it welded, as it were, their contents into a coherent whole, illustrating the splendors of the past, endocrinology appeared capable of insuring for the future much needed cohesion of our assets into a consistent, rational entity and, as a result, greatly increased efficiency in our humanitarian field.

The development of any such comprehensive change in our habits of thought is necessarily gradual and sometimes despairingly slow. But perhaps at no time has it been possible to illustrate more vividly than the present, the difference between endocrinology as it is generally interpreted by physiologists, and endocrinology studied in its

* Read before the Philadelphia Dental Society, March 27, 1923.

broad sense—that intended to assist all subdivisions of applied medicine, as interpreted from my viewpoint. Indeed, the chasm between us is so great that it has become necessary to identify two methods of endocrinological research: the physiological method and my own, which will be identified as the "all-science" method.

### THE PHYSIOLOGICAL METHOD

The most recent summary of endocrinology as interpreted by physiologists is one published but eight months ago by Prof. Swale Vincent,[1] of the University of London, in which he justly criticized many erroneous assertions and hypotheses which have tended to obscure and discredit it. Among these may be mentioned the prevailing custom of regarding as an internal secretion the extract of any organ despite the frequent warning of investigators that the results are pharmacodynamic only; the belief that all organs furnish an internal secretion; the view that the blood pressure and other standard conditions are maintained by a series of antagonistic chemical agencies; the very problematic vagotonia-sympathicotonia theory; the empirical use of organic products, etc.

Professor Vincent then reviews the present status of the main endocrins as interpreted by physiologists, his own branch of research. He concludes that "there is little or no evidence that the medulla of the adrenal bodies is of any importance;" that the function of these organs is "probably wrapped up in the activities of the cortex and not essentially connected with the formation of adrenin." "The thyroid gland," he suggests, "has been added to the animal organism in order to permit in it a greater range of flexibility of energy output." As to the parathyroids, recent work "seems to indicate that they control muscle tone by keeping down the amount of some toxic in the circulating blood"—probably guanidin. Concerning the pituitary body, recent work also "seems to indicate that many if not all results usually attributed to lesion of the pituitary body are really due to damage to the base of the brain,'' remarking in this connection, that ''if this is the case, we shall be forced to a reconsideration of our whole attitude in relation to the pituitary body.'' Recent literature suggests that the function of the thymus is antitoxic ''while there seems to be no reliable evidence that *mors thymica* is due to abnormality of the thymus.'' Finally, the evidence that in the gonads, the interstitial tissue, and in the pancreas, the islets of Langerhans provide an internal secretion is deemed ''by no means convincing.''

An impartial analysis of this showing by so competent a physiologist in a field to which he has given special attention, can not afford much food for thought. Indeed, the paucity of results is striking; nothing as regards the adrenals and nebulous possibilities with respect to the six other organs. When we recall that this represents the residue of the sixty-seven years elapsed since Brown-Séquard inaugurated the physiological era of edocrinology we, who bear the responsibility where suffering prevails and where death threatens, can not but suspect that there is something wrong, something paralyzing, in physiological methods of research.

Professor Vincent pleads for ''greater scientific discrimination than has hitherto been the case,'' but certainly no one has ever prevented physiologists from exercising to the fullest extent the highest degree of discrimination possible, and it would seem as if this full liberty of action and the laboratories and equipment at their disposal should have enabled them to discover the functions of at least *one* endocrine organ! But we have seen that such has not been the case. Indeed, if we took out of text-books of physiology today what clinicians, surgeons, biochemists and pharmacologists have contributed to the subject they would contain practically nothing. Nor can we but wonder whether undue self-sufficiency has not impaired the judgment of present-day physiologists when we find the brilliant endocrinological labors of such physiologists as Brown-Séquard, Schiff, Cyon, Langlois, Schâfer, Bayliss, Starling, Cannon and many others simply cast aside as unable to stand their inquisitorial discrimination. With such masters all wrong, physiology would no longer merit the confidence of thinking men.

The evil influence of this state of things crops up on all sides. Even the figments of imagination and gratuitous conclusions, justly criticized by Professor Vincent, would never have come to light, for they are all, directly or indirectly, traceable to efforts to compensate, by guesses or more or less inadequate studies, for what light physiology has failed to furnish. The prevailing random use of organic extracts is also due directly to physiological shortcomings, for it is obviously impossible to administer any such agents rationally while their mode of action—which at least in some instances, the adrenals and thyriod, for example, represent their function—remains unknown.

In the great fields of research, medical and surgical, the dicta of the physiological method are fatal to progress. The late Prof. H. S. Halsted, of Johns Hopkins, in an effort to solve an important surgical problem a few years ago was led to conclude that "it must be evident to everyone that there reigns the greatest confusion on the subject of the internal secretions." Another Johns Hopkins observer, Dr. J. R. Sprunt, who attempted recently to chart the symptoms of the various diseases of the endocrins *per se*, in which the prevailing physiological foundation is taken as standard, found it necessary to recall what he characterized as "the present confused state of knowledge concerning the normal functions of the endocrine glands."

Gentlemen, facts and results clearly indicate that the physiological method of endocrinology has totally failed to meet our needs. Is this due to want of sincerity on the part of its adepts? I only introduce this question to answer it negatively with due emphasis. The very frankness of Professor Vincent betokens the contrary. But this very fact brings us face to face with the skeleton in the closet, which is, as I urged twenty years ago, that physiology as interpreted by physiologists is utterly inadequate to develop endocrinology alone. Physiology itself, in fact, is being discredited by its adepts, whereas interpreted in its true light, as a contributor to our aggregate of knowledge along with other branches, it preserves in its entirety the lofty position to which it is entitled. This is where the all-science method comes in.

### THE ALL-SCIENCE METHOD

While the trend of the physiological method is ultradiscriminative and destructive, that of the all-science method, which I introduced over two decades ago, and which means the participation of all branches of biology, pure and applied, in the study of any medical question, is eminently broad and constructive. It eliminates individual discrimination with all its temperamental moods and prejudices, by permitting no single branch, physiology for instance, to predominate as arbiter over all others. Data contributed by all branches are co-ordinated, analyzed and synthetized, and whatever deduction or postulate is thus reached is not an arbitrary one as is now often the case; nor a preconceived theory, but the product of a mosaic, as it were, of all scientific data available on the subject. Physiology, clinical medicine, surgery, histology, pathology, pharmacology, biological chemistry, zoology, botany and all the various specialties, thus represent as many spokes in the wheel of progress. The value of a given contribution is not gaged by the prominence of the observer—for Pasteur, Lister and all great men had their days of obscurity,—but by its actual value and the manner in which it merges in with others equally well worked out. Apparently discordant data, often prove not to be such, for, if sound, they usually find in other branches of knowledge one or more links which convert them into valuable assets. Thus developed endocrinology destroys nothing of acquired medical knowledge, because it aims to do justice to all serious workers while adding to our storehouse of information and elucidating questions which for decades have remained obscure.

To avoid undue length of this paper, I shall illustrate the all-science method by analyzing a single endocrine structure, the adrenal medulla, and then summarize briefly the functions of the other endocrine organs also studied by this method. Brief remarks on the bearing they may have upon dental nutrition and caries will then be made.

### THE ADRENAL MEDULLA AND ITS SYSTEMIC ADNEXA AS THE MECHANISM OF TISSUE OXIDATION

Beginning with the adrenals, Swale Vincent concludes, we have seen, that there is little or no evidence that their medulla is of any importance. And yet, as shown by the "all-science" method, this portion of the adrenals supplies all the elements of the ultimate solution of a problem of commanding importance in *all* normal and morbid processes. As far back as 1895, Sir Michael Foster, one of the authoritative physiologists of his time, wrote: "We can not trace the oxygen through its sojourn in the tissues. We only know that sooner or later it comes back combined in carbonic acid." Twenty-six years later, in 1921, no progress had evidently been made in the elucidation of this problem, for as Professor Halliburton of London says in the last edition of his "Text-book of Physiology":[2] "Our knowledge of tissue respiration is so scanty that we can say little about its pathologic bearing."

The whole subject of respiration is no less obscure. The diffusion doctrine, still taught in textbooks of physiology, has been known to be defective ever since the middle of the last century, when many physiologists, including Robin,

Verdeil, Garnier, Duval, Vulpian, in France; Haldane and Lorrain Smith, in England, and notably Bohr, of Denmark,[3] found that the normal oxygen tension in arterial blood was often higher than the alveolar air. The Anglo-American Expedition to Pike's Peak in 1911 also observed that often at this high altitude, the oxygen tension in the blood became considerably higher than that in the alveolar air. While Haldane and the Oxford school explained this phenomenon by attributing to the lungs the power to clutch the oxygen and, through the alveolar epithelium, force it into the blood, Bohr and Henriques [4] held that the process required a substance "having a greater avidity for oxygen than the blood itself," . . . "a kind of internal secretion." The nature of this internal secretion, however, they failed to determine.

In 1900, various chemical, anatomical, physiological, biological and clinical facts led me to believe that the adrenal product might be the "internal secretion" sought by Bohr to meet the needs of the respiratory process. A comprehensive study of each phase of the process on the all-science plan led me (1903)[5] to the postulates: (1) That "the relative tension of oxygen in the blood and in the alveolar air is not a ruling factor in the process of respiration;" (2) that the secretion of the adrenals "which leaves these organs through the suparenal veins, is mixed with the plasma of the venous blood in the inferior vena cava;" (3) that "when the venous blood reaches the pulmonary alveoli, the marked affinity of the adrenalized plasma for oxygen causes it to absorb this gas from the alveolar air;" (4) that "the red corpuscles, after this operation, bathe in an oxygen-laden medium, and their hemoglobin becomes reconverted into oxyhemoglobin."

The work done by investigators in various branches: physiologists, physicians, surgeons, biologists, pathologists, biochemists, etc., since, even though absolutely sterile in the hands of the physiologists we have seen, has served to sustain this interpretation in all directions, aiding indirectly also to develop it. They found, for instance, that the active principle of the adrenal medulla, adrenalin or epinephrin, could, in doses as small as 1 milligram, subcutaneously or intramuscularly, increase the intake of oxygen and the output of carbon dioxid.[6, 7, 8] The respiratory rate was not only augmented,[6, 7, 8] but it was also found [9] that adrenalin caused prompt dilation of the bronchioles when they were contracted. Both minute and large doses of adrenalin were also found [10] to evoke an increase in the depth of respiration, and to cause [11] an increase of heat production of the respiratory quotient of the ventilation rate and of the respiration rate. Biology also contributes confirmatory evidence. H. H. Dale,[12] for instance, recalls that Abel "had found adrenin in relatively enormous quantity in the skin glands of the toad, where," he remarks, "it would seem to serve no possible function and might easily be regarded as a waste product." With the adrenal secretion as an active participant in the respiratory process, the presence of adrenin in the skin of these batrachians meets a self-evident need in view of the familiar fact that the skin forms part of their respiratory apparatus.

With respect to my conclusion that the adrenal secretion enables the hemoglobin to become converted into oxyhemoglobin on exposure to the alveolar air, investigators subsequently [13] found that adrenalin could itself act as does hemoglobin, and also [14] that blood from the adrenal vein, which transfers the adrenal secretion to the vena cava, invariably assumed a bright red, arterial color in from one to twenty minutes after dilution with salt solution, while blood from other organs, treated in the same manner, showed no change. Again, having added adrenalin to diluted human venous blood, they also [15] found that it caused an increase in the intensity of the oxyhemoglobin absorption-bands, "very strong evidence that it is adrenalin which is responsible for the increased amount of oxyhemoglobin found in the adrenal vein blood.'' The action of the adrenal secretion on metabolism is shown by the fact that its preparations even in small doses raise the temperature,[16, 17] while tumors composed of adrenal tissue do likewise.[18] Adrenal grafts may provoke such a rise of temperature, in fact, as to produce death.[19]

These few confirmatory facts from among a large number available, speak strongly through the manner in which they all merge in favor of the adrenals as the source of the substance which takes up the oxygen from the pulmonary air and carries it to the tissues. They do more, however; they sustain instead of discarding the well-grounded labors of Oliver and Schäfer, that suprarenal extracts can raise the blood pressure and increase cardiac dynamism—the latter previously noticed and recorded by Brown-Séquard; R. G. Hoskins' observation that small doses of adrenalin can lower the blood pressure; Cannon's emotional strees theory; Abelous and Langlois' antitoxic theory, and other less prominent views. But it establishes their true identity as *subsidiary phenomena* in which the *fundamental function* I attribute to the medulla plays the primary rôle in respiration and tissue oxidation, working in conjunction, we shall see, with another adrenal product.

Interpreted as the source of the systemic purveyor of oxygen, the adrenal medulla becomes a great central source of dynamic energy in so far as this element is concerned. Besides the adrenal medulla, however, reinforcing plants exist, so to say, in all parts of the body. Among these may be mentioned the many ganglia of the sympathetic nervous system and its paraganglia which consist of the carotid glands located where the common carotid arteries bifurcate, the tympanic glands in the auditory apparatus and an elongated body extending in the embryo from the adrenals to the bifurcation of the aorta. The sympathetic plexuses, such as the stellate, solar, renal, etc., also contain adrenal medullary substance and are usually, as are the ganglia, connected with the nerves *per se*. All of these, as I pointed out in 1903, confirmed by others since, are themselves channels for adrenoxidase-laden plasma which gravitates into their axis cylinders as does tetanotoxin. From my viewpoint, all this aggregate of adrenal medulla, ganglia, paraganglia plexuses and axis cylinders constitute the great system of oxygen distribution.

### THE ADRENAL CORTEX AND MEDULLA, AND THE ADNEXA AS THE MECHANISM FOR THE LIBERATION OF HEAT ENERGY

One of the disturbing enigmas of endocrinology is certainly the function of the cortical portion of the adrenals which constitutes nine-tenths of the whole organ. Swale Vincent refers to it, we have seen, as probably the seat of all adrenal activities. As interpreted from my viewpoint, it is the coworker of its mate, doing with another element, phosphorus, what the medulla does with oxygen—a postulate which I only submit tentatively, since I am now engaged in its study.

A peculiarity of the adrenal cortex is its wealth in lipoids, *i. e.*, fatty bodies, which give it a yellowish instead of the reddish color which characterizes the medulla. This total dissimilarity from the medullary portion and the fact that the corresponding tissue in many lower vertebrates is some distance apart from the true adrenals (being called in the former the interrenal tissues), suggests that they carry on entirely different functions. It is indeed known to be the seat of intense cellular activity and to be traversed by considerable blood. Its lipoids, of the many kinds that have been identified, include mainly two active bodies, lecithin, which is rich in phosphorus, and cholesterin or cholesterol, a monatomic alcohol, which contains no phosphorus.

Lecithin is also found in the brain, nerves, roe, egg yolks, sperm, the electric organs of fishes, milk, muscles, plasma, lymph, bile, etc. In the adrenal cortex its relative proportion appears to increase, in the fats it contains, with the muscular vigor and power of the animal; thus, while the proportion in man is about 2.08 per cent., in the horse it may be as high as 6.77 per cent. Again, lecithin has been found to act as a powerful stimulant of the heart muscle and to promote body growth in some. Individuals are occasionally met in practice in whom this growth process attains striking proportions, due to the development in the adrenal cortex as a tumor known as hypernephroma. Children suffering from such may in a short time grow to several times their normal size and show a corresponding mental and sexual development. They constitute the so-called "infant Hercules" of shows. In females, however, there is a marked tendency to reversal to the male type in every particular.

In the development of the brain and nervous system the adrenal cortex, owing mainly to its lecithin, plays a rôle of inestimable importance. Thus, anencephalic monsters invariably show absence of the adrenal cortex. Some doubt prevailed for a time concerning the presence of lecithin in the brain, but the recent labors of Levene and Ida P. Rolf,[20] at the Rockefeller Institute, have isolated and identified it in this organ, its composition being the same as that from the egg yolk, thus confirming the original observations of two French biochemists, Vanquelin (1812) and Gobley (1846), who introduced the term "lecithin," from the Greek "lekithos," which means egg yolk.

So far in my writings and pending the development of the lipoid question which was initiated only in 1901 by Overton, I attributed most effects, clearly due to phosphorus, to that of the cell nuclei which are also rich in this element and present in all tissues. Lecithin proper had long been thought to play a most important part in the processes of vital activity by its universal presence in all living cells, but the studies accumulated by biochemists on lipoids in general having been contradictory, particularly in respect to the investigations of Thudicum, it was deemed advisable to wait before developing the subject from the all-science standpoint. Briefly, as far as I have gone, *the phospho-lipoids appear to co-operate with adrenin for the elaboration of the systemic heat energy* We thus have in the adrenals the fundamental mechanism of the vital process, or rather its two paramount components. They can be traced everywhere together in both plants and animals.

But we have seen that the adrenal cortex contains another fat, cholesterin. Here, however, I must recall that we are not dealing with one containing phosphorus, but with a terpene derivative, thought formerly to occur only in plants as phytocholesterin. The same dual presence of lecithin and cholesterin observed in the adrenals can be followed out in all tissues and their cells, the blood corpuscles, the nervous system, where they are constituents of the myelin, the heart, the testicular interstitial tissue, etc. In some structures they accumulate in such quantities that the area containing it is like the adrenal cortex itself, yellow—the corpus luteum of the ovary for example.

The thymus also belongs to the phospho-lipoid series, particularly so in the infant; the occurrence of idiocy in individuals in whom this organ is lacking, the dwarf-like atrophic bones in pups deprived of this organ, and the defective fixation of lime salts associate it closely with calcium metabolism.

Moreover, just as we found substations of medullary adrenal oxidizing substance in all directions, so do we find substations of the adrenal cortical tissue throughout the body, all built up, as in the case of the latter, of columns of lipoid-containing cells in zones. They may be found on the surface of, or in, the kidney, where they sometimes develop into a type of tumor known as hypernephroma, as we have seen they do in the adrenals proper; also in the walls of, or between, blood vessels, both arteries and veins; in the liver, pancreas, peritoneum, broad ligament, epididymis, etc., and in any tissue in close relationship with the sympathetic nerve plexuses.

Next to the cortical portion of the adrenals in importance as a seat of lipoid droplets, however, is the anterior lobe of the pituitary. They are so numerous here, in fact, that when appropriately stained and examined microscopically, a section recalls the face of a confluent smallpox patient. Its leukocytes, particularly the eosinophiles and their granulations, supply a colorless substance which acts as a nutrient for its working mate, the posterior lobe. This suggests that the anterior lobe is not a secreting gland. Indeed, the evidence that it is such has been growing steadily weaker with time. Acromegaly and gigantism, for instance, have been attributed to this supposititious secretion; but no amount of the organ administered therapeutically, any more than tethelin, will make a dwarf grow. As far as acromegaly itself is concerned, we see the disease localized, limited to one part of the body, a single extremity or even one or more fingers—phenomena which a secretion can not explain. Most authors concur also that it is by stimulating the posterior or neural lobe, through, from my viewpoint, an excess of activating lipoids the anterior drives into it.

### THE POSTERIOR LOBE OF THE PITUITARY BODY AS A NERVE CENTER

The posterior lobe has also been discredited as a secreting gland. As its embryological history and its true name, the *neural lobe,* indicate, it is a nervous organ. Its prolonged and fictitious identity as a secreting gland was mainly sustained by the activity of extracts prepared from it, the effects of which, as first shown by Howell, being mainly due to the presence of adrenalin in its tissues, a fact confirmed by color tests. The presence of the adrenal medullary product is accounted for by the fact that it is the seat of active oxidation. But the posterior lobe is also rich in lipoids which are likewise present, we have seen, in the myelin of nerves and brain tissue. Its structure, in fact, has been compared to that of the cerebral gray matter by various investigators, the most recent of whom is A. G. McArthur,[21] thus identifying it anew as a nervous structure.

Swale Vincent, we have seen, concluded his analysis of the pituitary body with the statement that recent work "seemed to indicate that many, if not all, results usually attributed to the pituitary body were really due to damage of the base of the brain," and that, if this was the case, "we shall be forced to a reconsideration of our whole attitude in relation to the pituitary body." This is what I have been urging for over twenty years. I then held, on the basis of all-science studies, that the pituitary body was an important nerve center which sent nerve fibers by way of the basal tissues, the medulla oblongata, the spinal cord and the sympathetic nerves to all other ductless glands and also to the kidneys, the purpose being to control the elimination through the latter of the wastes resulting from the metabolic activity of these organs.

As to the lesions which suggested the need of discarding the secretion theory, they were located precisely in the course of the nerve path I had described. They interfered with the impulses transmitted through it to the kidneys, for instance, and by thus paralyzing the renal arteries caused them to dilate and to admit an excessive flow of arterial blood into the secreting cells, thus causing excessive urination. To illustrate what the all-science study of such a process means, I may mention that I traced thirty cases of a rare disease, diabetes insipidus, in which autopsies had located precisely the cause. This covered the literature of thirty-five years. Ten of the lesions lay in the *posterior lobe itself* and in the others along the nerve path, which I termed the *hypophyseo-renal path,*

down to the kidney. Much collateral evidence by other observers has also indicated its presence. The same applies to nerve paths to the adrenals, the thyroids, the parathyroids and the thymus, all, from my viewpoint, *bona fide* ductless glands of the comparatively few organs of this class.

The pineal body is also credited by some with a secretion; but as stated by Schäfer, the physiological and clinical data here are so discordant that its functions are quite obscure. The only light thrown upon this organ by the all-science method is that the frequently recorded resemblance between the symptoms caused by pineal growths and disorders of the hypophysis is due to pressure, direct or indirect, of these growths upon the basal tissues traversed by hypophyseal nerve paths.

### THE THYROPARATHYROID GLANDS AND THE ADRENALS IN METABOLISM AND IMMUNITY

The thyroid has probably been more carefully studied in all its aspects than any other gland since Graves in 1841 first studied the disease which bears his name. And yet, all Swale Vincent can gather from all this labor is that "it may serve to permit a greater range of flexibility of energy output." Professor Howell, of Johns Hopkins,[22] states in the latest edition of his textbook that "no explanation has been furnished in respect to nutrition." Yet, we clinicians know that thyroid feeding or thyroid overactivity raises the basal metabolic rate, that it causes in myxedema an increased intake of oxygen varying from 20 to 75 per cent., and 10 to 40 per cent. in animals, and a corresponding output of carbon dioxid. In 1903-1907, I submitted that this was due to the fact that the thyroid hormone, by increasing the lability of the phosphorus in all nuclei—their lecithin I would now say—beginning with those in the fat cells, to the action of the adrenoxidase. A multitude of facts studied by the all-science method have pointed to this deduction as the true function of the thyroid hormone.

What is the purpose of this whole endocrine mechanism? It is first, *to supply heat energy to the organism at large,* but so governed as to serve all functions to the best advantage. Summarized, the mechanism is as follows: The *adrenals* and their accessory structures produce the substances which supply all tissues their oxygen (medullary *adrenoxidase*) and their phosphorus (cortical *lecithin*), the function of these two elements being jointly to supply heat energy. To regulate this reaction the adrenoxidase is secreted by the red corpuscles in quantities sufficient to meet the needs of the anabolic phase of metabolism, while *cholesterin,* also present in the corpuscles and secreted by them, is known to moderate, as in hemolysis, venom poisoning, etc., the action of lecithin. It is because of this latter fact that cholesterin has been thought to possess defensive properties.

The purpose of this heat energy is to carry on the tissue exchanges or cellular metabolism. But this process consists of two phases: anabolism, the building up of the cell, and catabolism, the breaking down of its used materials and their conversion into wastes which may be easily eliminated. Now it is in the latter process that, from my viewpoint, the *thyroid,* jointly with the *parathyroids,* takes part. By labilizing the phosphorus in these wastes, they also cause the liberation of heat energy, but in this connection for a second purpose: that of carrying on a defensive process not only against tissue wastes, but also to destroy any toxin or pathogenic organism which may be present in the tissues and also to include phosphorus in their make-up.

How this is done affords another proof of the economical methods employed by nature to accomplish great things. She utilizes in the tissues, to destroy wastes, toxins and certain other poisons and bacteria, precisely the same enzymes she uses in the gastro-intestinal canal to digest foods. As Professor Mendel, of Yale,[23] well says: "Enzymes are no longer thought of exclusively as agents of the digestive apparatus; they enter everywhere into the manifold activities of cells in almost every feature of metabolism." The mechanism itself is simple: As all biochemists know, digestive enzymes are active according to the temperature to which they are subjected, the laboratory maximum temperature of trypsin, the digestive ferment found in the phagocytes as shown by Metchnikoff, being about 104 degrees F. in the thermostat, but higher by a few degrees in the tissues. This interpretation has enabled me to explain the processes of fever, and of autolysis, its harmful result when fever is excessive—all problems which also had remained obscure.[24]

This constitutes, briefly, a process of immunity in which the active agents and their sources are known, a fact which does not apply to any of the interpretations of immunity now extant, including Ehrlich's side-chain theory. Levi and Rothschild,[25] of Paris, in their work on the thyroid, write in this connection: "Sajous has attributed among the functions of the thyroid body a rôle which he similates to that of opsonins and auto-antitoxins. Fassin, Stepanoff and Marbé have all confirmed the influence of the thyroid on the blood's asset in alexins and opsonins." The former conducted the experimental work at the Bacteriological Institute of Liége and the two latter at the Pasteur Institute. McCarrison[26] also concluded extensive experiments with the statement that "the thyroid gland contributes largely to the body's antitoxic and bactericidal functions."

### THE ALL-SCIENCE METHOD IN THE STUDY OF DENTAL NUTRITION AND CARIES

Applied to dental science the method described proved a source of deep gratification by demonstrating anew the value of a broad interpretation of all work done. I must confess that, to my regret, the dental branches had not been included in my studies, because their literature was not merged with ours as is the case with all other branches of medical practice, and it was overlooked. But this affords me the opportunity of paying high tribute to the class of information obtained, and of assuring you that I will not hereafter deprive myself of its potent aid. As a tyro on the subject, I sought information and was advised by Dr. Fischelis, Professor of Histology in the Philadelphia Dental College, to study Professor Hopewell-Smith's works,[27] which I did with so much profit that practically none other was consulted. Of course, errors of interpretation may have crept in, but should such be the case, please blame the tyro and not the master.

Beginning with *Nasmyth's membrane* or enamel cuticle, considerable uncertainty as to its rôle seems to prevail. It is extremely thin and quite vulnerable to trauma, prolonged friction, etc., and when once torn affords a nidus for bacteria. While bacterial enzymes can disintegrate it, its rôle in caries, it is stated, is not at present understood. In the light of the views I have submitted, dental investigators, as we shall see, have established, excepting perhaps the adrenal oxidizing ferment, all the chemical bodies required to identify the process as one of progressive autolysis of the enamel cuticle after it has been injured. The micro-organisms themselves contain phospho-lipoids and the digestive enzyme, in addition to their toxins. The one factor missing is oxygen, however. Of course, with present teachings as guide, it is difficult to understand oxidation without the red corpuscles. But with adrenoxidase secreted as droplets into the plasma and the former becoming the oxidizing agent of serum, many obscure phenomena become clear. Various exudations and secretions, the saliva, for instance, contain adrenoxidase or oxidizing ferment, even though no red corpuscles are present.

This can be shown by a test which I devised while studying the chemical identity of vitamin C last year.

This active agent proved to be adrenalin and adrenoxidase, which in vegetables, fruit, etc., I found to be tyrosin and tyrosinase.[28] The familiar guaiac test is well known to demonstrate the presence of an oxidase, but as there are many of these, it became necessary to find one that would be specific to blood and plant respiratory pigments. After much searching, I finally adopted what I termed the adrenalin-$H_2O_2$ test, using as staining fluids nine parts of distilled water to one of hydrogen peroxid in one bottle, and a 1:1000 adrenalin chlorid solution in another. Whatever fluid or teased plant tissue is to be examined is first treated to a few drops of the adrenalin solution then to the 1:3000 $H_2O_2$ solution. With this test, as used in one hundred and ten foods, I found it possible to determine the vitamin C value of each, as represented by its content of tyrosin. As these values corresponded with those of the biological tests in animals, we have good ground for belief that they are sound.

What this test does is simply to enact *extra corpore* the process of blood oxidation in the lungs. We have seen that venous blood under appropriate conditions could be converted into arterial blood by the addition of adrenalin on exposing it to the air, thus proving the correctness of my respiration theory. Now, in plant life, tyrosin corresponds with the constituent of hemoglobin which causes it to become converted into oxyhemoglobin. The vitamin C value of a plant, therefore, is represented by its tyrosin content and it becomes red in proportion. Now, the turnip, the victim of the old saying: "One can not draw blood from a turnip," can actually be shown to contain plenty of it, because it is rich in tyrosin. The adrenalin-$H_2O_2$ test causes it to become vivid red.

But so does saliva under the same test turn red, though less so, thus showing that it contains, like blood plasma, the respiratory enzyme. As such, it is able to act as catalyzer, which means that it can take up oxygen and crowd it, as it were, upon any substances capable of being oxidized. That it is probably bound up with ptyalin is suggested by various facts. Again, the presence of adrenoxidase is indicated by the brown pigmentation, which corresponds with the brownish hue of leaves and the bronzing of Addison's disease, which pigmentation is due to stagnation of the adrenal hormone in the subcutaneous tissues.

On the whole, it would appear as if we had in the saliva the oxidizing enzyme necessary to liberate, with the phospholipoid of micro-organisms present, enough heat energy required to raise the activity of the digestive enzymes of these organisms sufficiently to provoke a destructive process. Once under the enamel cuticle, this process progresses actively, the foregoing aggressive agents being probably obtained from dead organisms, as endotoxins, while the living organisms multiply actively.

With respect to the *enamel*, investigators seem to disagree as to the presence of living matter in the interstices between the rods, in the form of fibers intercommunicating by means of delicate fibrillæ which pierce the rods vertically; also as to the presence of living material throughout, some considering the enamel entirely inorganic. Yet the brown striæ of Retzius suggest the presence of adrenoxidase, while the continuation of the caries in the pits and fissures of the enamel and the loosening of the inter-columnar substance, seems to me to indicate that the proteolytic process described for Nasmyth's membrane merely continues here. Such being the case, the presence of the living fibrillæ and intercommunicating network would be the only tissues normally vulnerable to such a destructive process.

As to the *dentin*, we again meet with possible adrenoxidase carriers in the shape of the dentinal tubules, thought to contain protoplasm. Professor Hopewell-Smith in this connection, however, expresses the opinion that "a serous exudation fills the tubes, bathing the dentinal fibrils.'' I believe that the tests I have described would indicate the presence of the adrenal oxidizing ferment in them. You will recall that I referred to the presence of this substance in the axis cylinders of nerves, stating that since 1903, when I pointed out this fact, confirmatory evidence had been published in various directions. Mr. J. Howard Mummery is referred to by Professor Hopewell-Smith as having been able to stain nonmedullated fibers with methylene-blue. Now it is because the latter is oxidized that it assumes the blue color in the nerves.

Caries here proceeds as in the overlying tissues, only we obtain actual proof of the presence of the adrenal enzyme and of the phospho-lipoid. The former is shown by the presence of the brown pigmentation observed, and the latter by shining irregular granules, regarded by some dental authors ''as fats,'' which you can readily see in red corpuscles when examined by the ultramicroscope. As they secrete their contents in the plasma (as blood platelets), they are doubtless present in the dentinal tubules also. As to the necessary enzyme, Professor Hopewell-Smith refers in this connection to the ''peptonizing action of bacteria'' as extending the damage, etc.

Of course, I have not touched upon the many theories with which you are familiar. The above merely suggests a pathogenic process which I found to prevail in many destructive disorders, and of which all the constructive factors seem to be present in dental tissues.

A few words upon the influence of the *endocrinopathies* upon dental caries will close this, I am afraid, overlong address. Those of an adynamic type, cretinism, myxedema, Addison's disease, etc., are familiar predisposing factors, owing to the deficient nutrition, calcium metabolism, vulnerability to infection, local and general, they entail. Perhaps the gravest expression of this effect on the teeth is scurvy, which disease is prevented by vitamin C, to which I previously referred as tyrosin in plants and adrenalin in animals. In this disorder there is gradual tissue death. Concerning the pituitary body, you will recall that I referred to this organ as a co-ordinating center which sends nerves to various endocrins and other tissues. I may recall in this connection a prophetic remark of Dr. E. C. Kirk,[29] who, referring to various reflex disorders nine years ago, wrote that "it requires no great stretch of the imagination to realize the possible, even probable, disturbance of the pituitary body in cases of pathological dentition." Among the organs under pituitary influence is the pancreas which, besides its proteolytic ferments, secretes amylopsin, an enzyme capable of converting 40,000 times its weight into dextrose and maltose. Dr. Kirk rightly recalls in this connection the labors of Goetsch, Cushing and Jacobson, with relation to the influence the posterior lobe of the pituitary exerts upon carbohydrate metabolism. I would recall in this connection that ptyalin, which acts similarly to the amylopsin, is probably bound up with the adrenal oxidizing ferment. This salivary enzyme might be a factor in promoting the sensibility to caries to which Dr. Kirk refers.

In *calcium metabolism*, so actively influenced by the thyroid and thymus, we probably have the explanation of the fact that youth is the period of greatest susceptibility. The thymus being a subsidiary organ calculated to contribute to the body's increased assets of phospholipoids through its thymocytes, during the development of the osseous and cerebro-spinal systems, any deficiency on its part up to about the sixteenth year compromises the solidity of the dental structure, which includes 66 per cent. of calcium phosphate. When adult age is reached the calcium equilibrium is well established and the tendency to caries is greatly reduced. Pregnancy is also known to predispose to caries. This is clearly explained by the demonstrated fact that enormous quantities of phospholipoids are appropriated by the fetus at the expense of the maternal supply, thus inhibiting correspondingly, unless adequately replaced by appropriate foods, the needs of her dental osseous tissues.

In *infections* we have another frequent cause of caries. Syphilis does its main harm, in my opinion, through its debilitating influence upon the endocrine glands, and in tertiary cases the therapeutic use of thyroid gland—the active principle of which is iodin in organic combination, thus accounting for the efficiency of potassium iodid—is sometimes remarkably efficacious. In hereditary syphilis in which caries is frequent, the rôle of the thyroid is so marked that treatment during pregnancy may overcome the evil influence of the disease on the offspring. In a family in which three idiotic children were traced to luetic infection, personal treatment during a fourth pregnancy procured a normal child. Tuberculosis, which also favors caries, is known to deplete actively the body of its phospholipoids. The caries brought on by acute infections is not only the result of a similar state but also of the exhaustion of the various endocrins. As we have seen, the adrenal medulla and cortex sustain the defensive oxidation which renders the bacteriolytic and antitoxic enzymes effective in conjunction with the sensitizing action of thyroid hormone. All these organs are debilitated for a time after convalescence and favor the necrotic dental process.

In closing, permit me to reiterate that the foregoing is submitted only tentatively, in the hope that what the biochemistry of the endocrins seems to suggest in the tissues at large will contribute somewhat to the elucidation of obscure questions, which seem also to exist in your special line of research. Whether the dental structure presents the same characteristics as other osseous tissues will be for you to decide, for I do not feel competent to do so. It does seem, however, that the principle of co-operation on the lines I have described would serve us all advantageously in the development of all lines of medical research, including your own.—*Dental Cosmos.*

REFERENCES

[1] VINCENT, SWALE: Arris and Gale Lecture. *Lancet*, 1922, cciii, p. 313.

[2] HALLIBURTON: "Text-book of Physiology." 1911, 10th Edition, p. 392.

[3] BOHR, C.: *Archiv f. Physiol.* 1891, ii, p. 236.

[4] BOHR AND HENRIQUES: *Arch. de Physiol.* 1897, ix, p. 459.

[5] SAJOUS, C. E. DEM.: "Internal Secretions and the Principles of Medicine." Philadelphia, 1st Edition (1903), pp. 127 and 801-850. Same in 2nd (1907) and 3rd (1909) Editions. In 4th (1911), 5th (1912), 6th (1914), 7th (1916), 8th (1919), and 9th (1921) Editions, pp. 56-70 and 801-850. In 10th (1922) Edition, pp. 2-8, 59-71 and 801-850. Also *Gaz. des Hôp.* (Paris), 1907, lxxx, p. 1407; *Practitioner* (Lond.), 1915, xciv, p. 179.

[6] BYELAVENTZ, P. P.: *Rousskii Vratch*, ii, p. 247, 1903.

[7] BERNSTEIN AND FALTA: *Verhandl. d. deut. Kongr. f. inn. Med.*, 1912, xxxix, p. 536.

[8] TOMPKINS, STURGIS AND WEARN: *Arch. Internal Med.*, 1919, xxiv, p. 269.

[9] JACKSON, D. E.: *Jour. Pharm. and Exper. Therap.*, 1913, iv, p. 291.

[10] NICE, ROCK AND COURTRIGHT: *Amer. Jour. Physiol.*, 1914, xxxiv, p. 326.

[11] SANDIFORD, I.: *Amer. Jour. Physiol.*, 1920, ii, p. 407.

[12] DALE, H. H.: *Proceedings* Royal Soc. of Med.; Therap. and Pharm. Sect., Lond., vii (3), p. 34, 1913-1914.

[13] KARIYA AND TANAKA: *Jour. Tokyo Med. Assoc.*, 1913, xxvi, No. 20; Abst. in *Sei-I-Kwai Med. Jour.*, 1913, xxxii, p. 10.

[14] MENTEN AND CRILE: Cited by Menten: *Amer. Jour. Physiol.*, 1917, xliv., p. 176.

[15] MENTEN, M. L.: *Ibid.*

[16] LEPINE, R.: *La Semaine Méd.*, 1903, xxiii, p. 53.

[17] MOREL, L. E.: *Le Progrès Méd.*, 1903, xxxii, pp. 63, 81.

[18] ISRAEL, J.: *Deut. med. Woch.*, 1905, xxxi, p. 1745.

[19] COURMONT, PAUL: "Titres et Travaux Scientifiques," Lyon, 1907, p. 90.

[20] LEVENE AND ROLF: *Jour. of Biol. Chemistry*, 1921, xlvi, p. 453.

[21] MCARTHUR, A. G.: *Jour. Amer. Chem. Soc.*, 1919, iv, p. 1225.

[22] HOWELL: "Text-book of Physiology," 1918, 7th Edition, p. 878.

[23] MENDEL: *Jour. Amer. Med. Assoc.*, 1906, xlvi, p. 843.

[24] SAJOUS, C. E. DEM.: *Amer. Jour. Med. Sci.*, 1922, clxiv, p. 625.

[25] LEVI AND ROTHSCHILD: "*Physiol. de la glande thyroide*," Paris, 1910.

[26] MCCARRISON, R.: *Lancet*, 1913, clxxxiv, p. 365.

[27] HOPEWELL-SMITH, A.: "Dental Anatomy and Physiology," London, 1913; "Normal and Pathological Histology of the Mouth," 2nd Edition, 1918.

[28] SAJOUS, C. E. DEM.: *N. Y. Med. Jour.*, 1923, cxvii, p. 325.

[29] KIRK, E. C.: *Dental Cosmos*, 1914, lvi, p. 1.

